# Highly efficient release of simvastatin from simvastatin-loaded calcium sulphate scaffolds enhances segmental bone regeneration in rabbits

**DOI:** 10.3892/mmr.2014.2101

**Published:** 2014-04-01

**Authors:** XIN HUANG, ZHONGMING HUANG, WEIXU LI

**Affiliations:** Department of Orthopedic Surgery, The Second Affiliated Hospital, School of Medicine, Zhejiang University, Hangzhou, Zhejiang 310009, P.R. China

**Keywords:** simvastatin, calcium sulphate scaffold, recombinant human bone morphogenetic protein 2, bone regeneration

## Abstract

A number of clinical and experimental studies have investigated the effect of simvastatin on bone regeneration. In the present study, the release of simvastatin from simvastatin-loaded calcium sulphate (CS) scaffolds and the effect of these scaffolds on osteogenic differentiation of bone marrow-derived mesenchymal stem cells (MSCs) *in vitro* and the effect of simvastatin locally applied from CS scaffolds on bone regeneration were investigated. A total of 26 complete 1.2-cm bone defects were created in the ulna of rabbits, which were treated with CS, simvastatin-loaded CS or recombinant human bone morphogenetic protein 2 (rhBMP)-2-loaded CS. Simvastatin was highly efficiently released from simvastatin-loaded CS at the onset and stable release was maintained. Alkaline phosphatase was highly expressed in the MSCs co-cultured with simvastatin/CS scaffolds for 7 and 14 days. The defects treated with rhBMP-2-loaded CS and simvastatin-loaded CS showed significantly higher X-ray analysis scores and a larger amount of bone formation as determined by histology compared with the CS group (P<0.05). No significant differences in the X-ray score and bone formation were observed between groups with rhBMP-2-loaded CS and simvastatin-loaded CS (P>0.05). Simvastatin is capable of promoting osteogenic differentiation of MSCs *in vitro* and stimulating bone regeneration when locally released from CS scaffolds into bone defects. The beneficial effect of simvastatin was similar to that of rhBMP-2. In conclusion, the present study suggested that the simvastatin-loaded CS scaffolds may have great potential in bone tissue engineering.

## Introduction

The repair of large bone defects, caused by injury, eradicated tumor masses or progressive periodontal diseases is challenging. Autologous bone grafts, allografts and alloplasts have been used for bone repair (1). Autologous bone is considered the gold standard of graft materials (2,3). However, it has a number of shortcomings, including morbidity of the donor site and limitation of the amount of bone that may be harvested (4,5). Allografts and alloplasts may cause immunologic responses and endemic risks. Therefore, it is necessary to identify different types of bone substitutes.

Among the numerous bone substitutes, calcium-sulfate (CS) is safe, highly biocompatible, bioresorbable and osteoconductive (6,7). In addition, its potential as a good carrier for local release of antibiotics and growth factors has been demonstrated (8–12). The osteogenic properties of bone substitutes may be enhanced when combined with osteoinductive substances, including recombinant human bone morphogenetic protein 2 (rhBMP-2). However, rhBMP-2 is expensive, limiting its clinical application. Notably, simvastatin, a cholesterol-lowering drug, has been shown to stimulate new bone formation in murine calvaria and also increase bone volume when administered orally to rats by induction of BMP-2 (13).

Thus, simvastatin-loaded CS may be attractive as a novel bone substitute enhancing bone regeneration. In the present study, the release of simvastatin from simvastatin-loaded CS scaffolds, the effect of simvastatin on the osteogenic differentiation of bone marrow-derived mesenchymal stem cells (MSCs) *in vitro* and the effects of simvastatin-loaded CS on the regeneration of segmental bone defects in the ulna of rabbits were investigated.

## Materials and methods

### Fabrication of simvastatin-loaded and rhBMP-2-loaded CS scaffolds

Osteoset^®^ (Wright Medical, Arlington, TN, USA), a medical-grade CS powder, was used in the present study. Simvastatin (Sigma-Aldrich, St. Louis, MO, USA) was dissolved in 75% ethanol at a concentration of 100 mg/ml. rhBMP-2 (Genescript, Piscataway, NJ, USA) was dissolved in phosphate-buffered saline (PBS; Gibco Life Technologies, Grand Island, NY, USA) at the concentration of 1 mg/ml. For the preparation of simvastatin-loaded and rhBMP-2-loaded CS scaffolds, 0.48 g CS powder, 0.145 ml distilled water and 5 μl simvastatin solution or 0.48 g CS powder, 0.14 ml distilled water and 10 μl rhBMP-2 solution were aseptically mixed in a dish. The mixture was transferred into a circular mold of 4-mm diameter and 12-mm thickness to create cylinders for implantation. In addition, to compare the differences in simvastatin release between CS scaffolds of different weights at the same dose, 0.16 g CS powder, 0.045 ml distilled water and 5 μl simvastatin solution were mixed, then simvastatin-loaded CS scaffolds (0.16 g) of the same diameter were fabricated. Finally, 0.5 mg simvastatin was added to each scaffold.

### In vitro assay of simvastatin release from simvastatin-loaded CS scaffolds

The simvastatin-loaded CS scaffolds (160, 480 mg) were placed in 5 ml PBS (Gibco-BRL) at 37°C and the PBS was changed at 1, 3, 4, 6, 8, 11, 14 or 21 days, respectively. At every time-point, the solution absorbance was measured at a wavelength of 238 nm using an ultraviolet-visible spectrophotometer, while the simvastatin concentration was determined from a standard curve prepared with various amounts of simvastatin.

### MSC isolation from rabbit bone marrow and culture

Rabbits heparinized bone marrow (BM) cells were aspirated from the humerus with an 18-gauge needle. The mononuclear cells were centrifuged at 1,000 × g for 10 min at room temperature. The cells were collected and resuspended in low glucose Dulbecco’s modified Eagle’s medium containing 10% fetal bovine serum (Gibco-BRL, Carlsbad, CA, USA) and 1% antibiotics (100 U/ml penicillin and 100 U/ml streptomycin; Gibco Life Technologies). Following 48 h in culture, the medium was removed and fresh medium was added to each flask. Cells were maintained at 37°C in a humid atmosphere with 5% CO_2_ and the medium was changed every two days. When adherent cells reached 80–90% confluency, they were detached with 0.25% trypsin-EDTA (Gibco-BRL) and replated at a ratio of 1:3 in regular growth medium to allow for continued passaging. Only passage three cultures were used for the experiments.

### Osteogenic differentiation of MSCs stimulated by simvastatin in vitro

The osteogenic differentiation of MSCs cultured with simvastatin-loaded CS scaffolds was examined by measuring alkaline phosphatase (ALP) activity expressed by the cells. Scaffolds were first placed in a six-well plate and MSCs were seeded with samples at a density of 5×10^5^ cells/sample and cultured for 7 or 14 days in control medium. The medium was changed every two days. At 7 and 14 days, the medium was removed and the samples were transferred to a new plate, washed with PBS, followed by the addition of a cell lysis solution. The samples were then processed through two freeze-thaw cycles (−70°C and room temperature; 45 min each) to rupture the cell membrane and extract the proteins. ALP activity of the cell lysate was determined with a p-nitrophenyl phosphate (pNPP) phosphatase assay kit (Nanjing Jiancheng Bioengineering Co., Ltd., Nanjing, China). Total protein content of the cell lysate was measured using a bicinchoninic acid (BCA) protein assay kit (Nanjing Jiancheng Bioengineering), according to the manufacturer’s instructions. Dividing the quantity of ALP by the amount of total protein normalized the specific amount of ALP. ALP activities of MSCs cultured on CS scaffolds were used as controls.

### Animals and surgical procedure

A total of 18 New Zealand white rabbits (provided by the Laboratory Animal Center of Zhejiang University, Hangzhou, China) weighing 2.5–3.0 kg were used for the study. All animal experimental instructions were approved by the Animal Care and Use Committee of Zhejiang University (Hangzhou, Zhejiang, China) and followed the ‘Principles of Laboratory Animal Care’ (NIH publication No. 86–23, revised 1985), as well as specific national laws (e.g., the current version of the German Law on the Protection of Animals). The animals were anesthetized with an intravenous injection of 3% sodium pentobarbital (30 mg/kg). A total of 24 complete bone defects of 1.2 cm were created with a high-speed saw under irrigation with physiological saline and the periosteum was removed. The radius was left intact for mechanical stability.

The 36 defects were randomized into 3 groups (n=6 in each group) and treated with the CS scaffold (group A), simvastatin-loaded CS scaffold (group B) or rhBMP-2-loaded CS scaffold (group C, positive control). The rabbits of each group were sacrificed at 4 or 8 weeks following surgery.

### Gross observation

The status of bone repair and growth of callus were observed in samples removed through the original incision following animal sacrifice.

### Radiological examination

Anterior and posterior radiographs of the bone defects were obtained to observe bone healing four and eight weeks following implantation. Bone formation was assessed in each group following instructions with triple blinding according to the Lane-Sandhu X-ray scores (14) (Table I).

### Histological observation and histomorphometrical analysis

Samples were fixed with 10% paraformaldehyde, decalcified with formate-sodium formate and embedded with paraffin. Sagittal plane sections (7-μm thick) from the interface region of each implant were prepared and stained with hematoxylin and eosin (H&E), then examined under a light microscope (Olympus, Tokyo, Japan).

To quantitatively determine the amount of newly formed bone, the histological sections were statistically analyzed at four and eight weeks following implantation following the procedures described previously (15). Three pieces of histological sections of each sample were randomly selected. Following H&E staining, each section was observed by light microscopy (magnification, ×40) and at least 10 images were randomly obtained per section. Using the image analytical software Image-Pro Plus 6.0 (Media Cybernetics Inc, Acton, MA, USA), the amount of newly formed bone was expressed as the percentage of the newly formed bone area within the original drill defect area.

### Statistical analysis

Lane-Sandhu X-ray scores and newly formed bone areas were examined by one-way analysis of variance. Data analysis was performed using SPSS software (version 15.0; SPSS Inc., Chicago, IL, USA). Fisher’s Least Significant Difference test was used for multiple comparisons. P<0.05 was considered to indicate a statistically significant difference.

## Results

### In vitro release behavior of simvastatin from simvastatin-loaded CS

**Figure 1:**
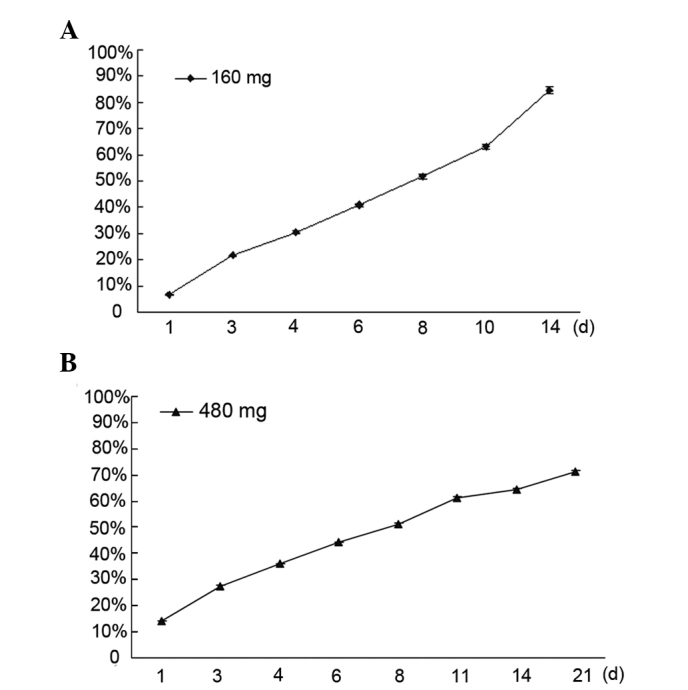
Cumulative release of simvastatin *in vitro*. (A) Simvastatin-loaded CS scaffold (160 mg). (B) simvastatin-loaded CS scaffold (480 mg). CS, calcium sulphate; d, days.

The *in vitro* release pattern of simvastatin from simvastatin-loaded CS is shown in Fig. 1. On day 1, ~5.2% and 11.3% of simvastatin was released from the simvastatin-loaded CS (160 and 480 mg), respectively, and a stable release was maintained. By day 14, ~85% of the loaded simvastatin was released from the simvastatin-loaded CS (160 mg). However, in the simvastatin-loaded CS (480 mg), ~65% of the loaded simvastatin was released by day 14 and 71% of the loaded simvastatin was released by day 21.

### ALP measurement

**Figure 2:**
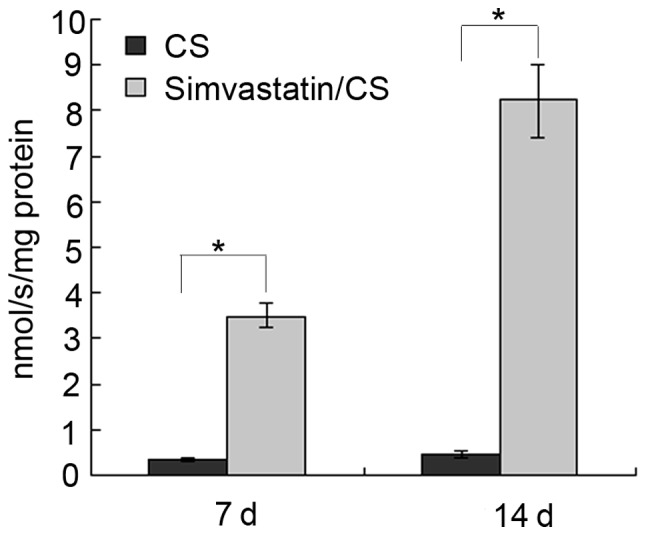
ALP expression of bone marrow-derived MSCs following co-culturing with simvastatin/CS for 7 and 14 days. ALP, alkaline phosphate; MSCs; mesenchymal stem cells; CS, calcium sulphate. ^*^P<0.05, CS vs. Simvastatin/CS.

Specific ALP expression increased on simvastatin-loaded CS scaffolds between 7 and 14 days (Fig. 2). At 7 days, ALP (mean ± standard deviation; n=5) levels were 3.51±0.28 nmol/sec/mg protein for MSCs on simvastatin-loaded CS scaffolds, which was higher than 0.35±0.03 nmol/sec/mg protein on CS scaffolds (P<0.01). At 14 days, ALP levels increased to 8.22±0.81 nmol/sec/mg protein in cells on the simvastatin-loaded CS scaffolds, which was significantly higher than 0.48±0.04 nmol/sec/mg protein for cells on CS scaffolds (P<0.01).

### Gross observation

All rabbits had normal diets and movement following surgery and survived until the scheduled date of sacrifice without any apparent complications.

**Figure 3:**
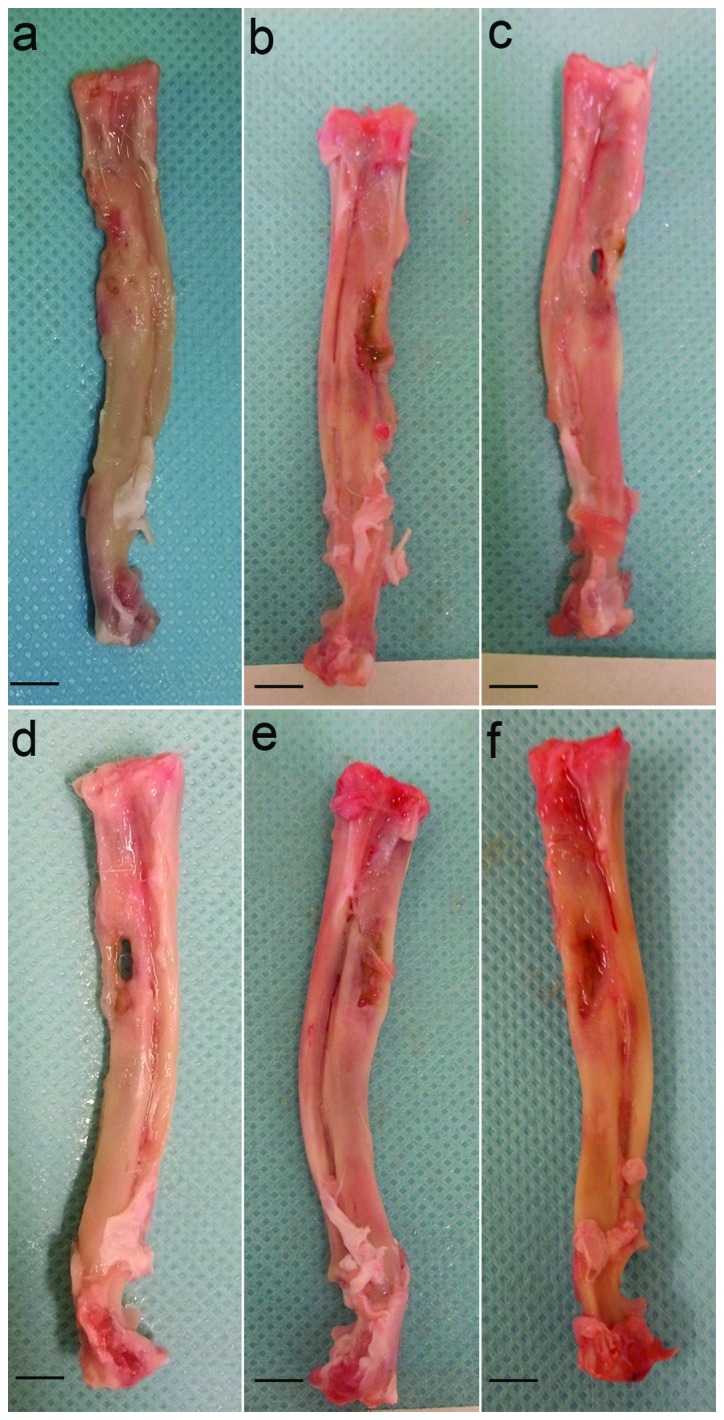
Gross observation of ulnar defects at (A–C) 4 and (D–F) 8 weeks following implantation. A and D, CS group; B and E, simvastatin-loaded CS group; C and F, rhBMP-2-loaded CS group; scale bar, 4 mm. CS, calcium sulphate; rhBMP-2, recombinant human bone morphogenetic protein 2.

At four weeks, the majority of defects in group A was filled with fibrous tissue (Fig. 3A). In groups B and C, new bone only formed in the extremities of defects and the areas next to the radius (Fig. 3B and C). The outside of the defects linked together in group C (Fig. 3C). At eight weeks, new bone formed in the inside of the defects in group A and the outside of the defects was filled with fibrous tissue. In addition, there was a cavity in the middle of the defect (Fig. 3D). In groups B and C, the ulna achieved bone union. However, they were not completely regenerated; only the inside and outside cortical bone was regenerated (Fig. 3E and F).

### X-ray examinations

**Figure 4:**
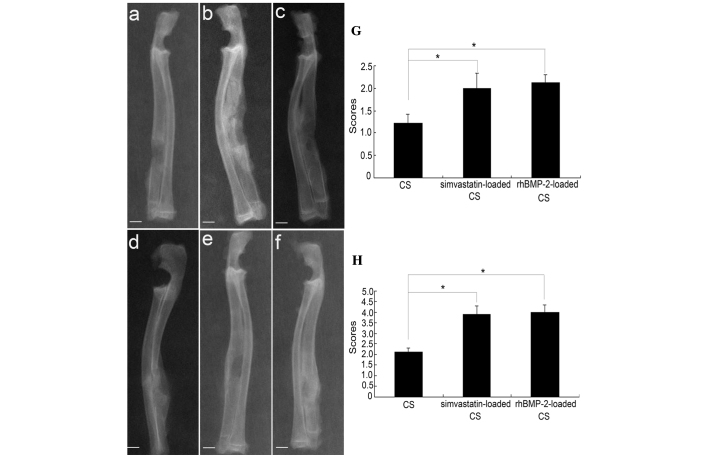
Radiographic analysis of ulnar defects at (A–C) 4 and (D–F) 8 weeks following implantation and Lane-Sandhu X-ray scores (G, 4; H, 8 weeks) in each group. A and D, CS group; B and E, simvastatin-loaded CS group; C and F: rhBMP-2-loaded CS group. Results are expressed as mean ± standard deviation. Scale bar, 5 mm; ^*^P<0.05, vs. CS. CS, calcium sulphate; rhBMP-2, recombinant human bone morphogenetic protein 2.

The X-ray images of each group are shown in Fig. 4. In group A, the bone defects were not repaired at four weeks (Fig. 4A). In groups B and C, the CS was mostly degraded at 4 weeks and osteoid tissue had formed at the extremities and areas next to the radius (Fig. 4B and C). At eight weeks, the CS was completely degraded. Osteoid tissue had formed in the areas next to the radius in group A (Fig. 4D). In groups B and C, the CS and bone tissue connected. The medullary cavity achieved partial recanalization; the inside and outside cortical bone was regenerated (Fig. 4E and F).

The bone formation scores were evaluated according to the Lane-Sandhu score standard. The X-ray scores increased with time and the score of group A was significantly lower compared with groups B and C at postoperative weeks four and eight (P<0.05). The scores of group C were slightly higher compared with group B. However, there was no significant difference between groups B and C both at four and eight weeks (P>0.05; Fig. 4G and H).

### Histological analysis

**Figure 5:**
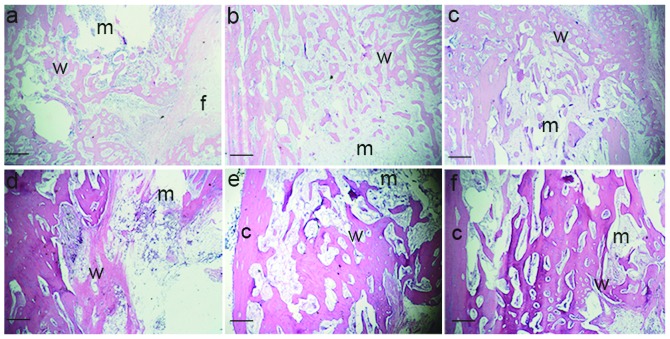
Histological examination of the repaired bone tissue at (A–C) 4 weeks and (D–F) 8 weeks following implantation. A and D, CS group; B and E simvastatin-loaded CS group; C and F, rhBMP-2-loaded CS group. H&E staining; magnification, ×20; scale bar, 200 μm. w, woven bone; c, cortical bone; f, fibrous tissue; m, medullary cavity; CS, calcium sulphate; rhBMP-2, recombinant human bone morphogenetic protein 2; H&E, hematoxylin and eosin.

At four weeks following implantation the CS was mostly degraded. In group A, small amounts of woven bone were observed to form in the extremities of the defects. The outsides of the defects were filled with fibrous tissue (Fig. 5A). In group B, abundant woven bone formed in the extremities of the defect areas next to the radius and marrow cavities (Fig. 5B). Compared with group B, the woven bone of group C was increased and denser, which was observed in the majority of defects, even on the outside (Fig. 5C).

At eight weeks, the two ends of the original ulna were united with the regenerated new bone. The CS was completely degraded. Compared with four weeks, the newly formed bone resembled normal cortical bone. In group A, dense woven bone formed in the extremities and inside the defect. However, the outside was not regenerated with bone and the marrow cavity was not recanalized (Fig. 5D). In groups B and C, the cortical bone regenerated on the inside of the defects and the outside was filled with abundant dense woven bone. In addition, the medullary cavity formed and achieved slight recanalization (Fig. 5E and F).

### Bone area analysis

**Figure 6:**
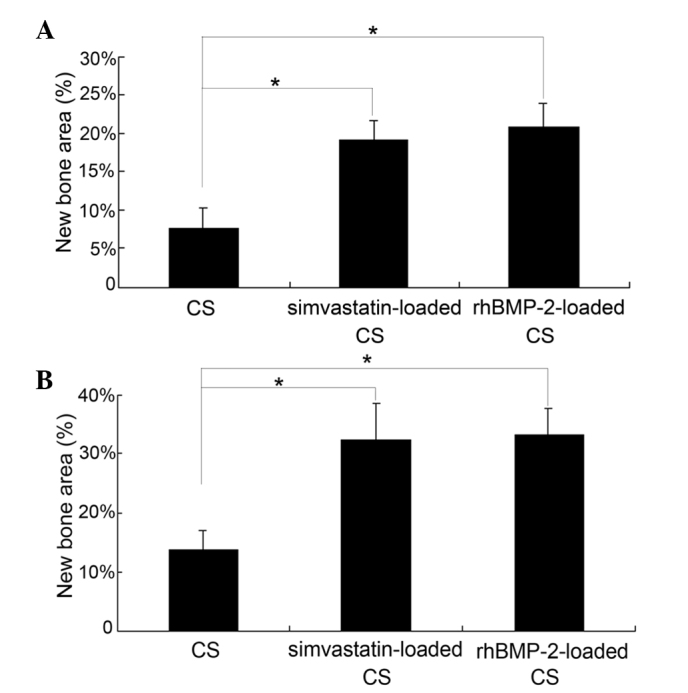
Comparison of the area of newly formed bone tissue in the defect area of three groups at (A) 4 and (B) 8 weeks following implantation. Results are expressed as mean ± standard deviation. ^*^P<0.05, vs. CS. CS, calcium sulphate; rhBMP-2, recombinant human bone morphogenetic protein 2.

The area of newly formed bone in groups B and C was significantly higher compared with group A at four and eight weeks following implantation (P<0.05). No significant difference in the area of the newly formed bone was observed between groups B and C at each time-point (Fig. 6).

## Discussion

The present study demonstrated that simvastatin was highly efficiently released from simvastatin-loaded CS, promoted the osteogenic differentiation of MSCs and stimulated bone regeneration when it was locally released from CS scaffolds into bone defects. The beneficial effect of simvastatin, locally applied from CS scaffolds, was similar to those of rhBMP-2.

In the present study, simvastatin was incorporated into CS scaffolds and was gradually released in a sustained manner. The release assay showed that the release efficiency correlated with the volume of simvastatin-loaded CS. Simvastatin-loaded CS (480 mg) showed a more efficient release of simvastatin on day 1 as compared with simvastatin-loaded CS (160 mg), which had a lower volume. On day 1, ~11.3% of the simvastatin was released from simvastatin-loaded CS (480 mg). However, from the simvastatin-loaded CS (160 mg), only 5.2% of the simvastatin was released. In addition, the release of simvastatin from simvastatin-loaded CS (480 mg) was extended over a longer time period as compared with the 160 mg sample. At day 21, 71% of simvastatin was released. In the simvastatin-loaded CS (160 mg), ~85% of the loaded simvastatin was released at day 14. The results suggested that the simvastatin-loaded CS (480 mg) may provide a longer period of simvastatin release, as well as a highly efficient release of simvastatin at the onset.

A previous study suggested that the appropriate concentration of simvastatin was 0.5–1 μM for *in vitro* culture and MSCs could not proliferate in a medium containing >2.5 μM simvastatin (16). Therefore, in the present study, MSCs were co-cultured with 1 μM simvastatin for 14 days and the osteogenic differentiation of MSCs was investigated through detection of ALP expression. The ALP expression of MSCs co-cultured with simvastatin was significantly higher, which meant that simvastatin was able to induce the osteogenic differentiation of MSCs. Similar results were observed in a previous study where human adipose tissue-derived stromal cells were treated with 0.01, 0.1 and 1 μM simvastatin (17).

The mechanism of sustained release of simvastatin occurs through degradation of CS. *In vivo*, through the degradation of CS, simvastatin was persistently and locally released to induce bone formation. Local application has a therapeutic advantage by preventing systemic side-effects. Previous studies have investigated the effects of locally applied simvastatin. In these studies, the doses of simvastatin used have been variable: 2.2 mg (18), 0.5 mg (19,20); 0.1, 0.5, 1.0, 1.5 and 2.2 mg (21) showed positive or negative effects on bone repair. In a study by Wong and Rabie (19), 0.5 mg simvastatin in an aqueous solution was added to a collagen matrix in calvarial defects in rabbits and a 308% increase in new bone was present in the simvastatin-collagen group compared with the collagen group alone at 14 days. Stein *et al* (21) found that 0.5 mg simvastatin appeared to be the optimal dose for single local application and a dose of 0.5 mg produced the best bone growth/inflammation ratio. Based on these findings, 0.5 mg simvastatin was selected and incorporated into the CS scaffolds for segmental bone regeneration.

Simvastatin could stimulate the BMP-2 expression in osteoblasts and inhibit the osteoclastic activity (22,23). Furthermore, a number of experimental animal studies have demonstrated a beneficial effect of simvastatin on bone formation (19,20,24,25), which is in agreement with the results of the present study. The simvastatin-loaded CS group had a significantly larger bone area compared with the CS group at four and eight weeks. CS not only worked as an osteoconductive scaffold for bone regeneration, but also as a carrier for releasing simvastatin. The released simvastatin could promote osteoblastic differentiation of bone marrow-derived MSCs, which was confirmed in a previous study (26). Another possible reason for the effects of simvastatin on bone regeneration may be their effect on angiogenesis and VEGF expression (27). In a study by Wong *et al* (28), simvastatin triggered the early expression of growth factors, including VEGF and BMP-2, and induced and accelerated formation of bone locally (28).

Notably, the present study revealed that simvastatin stimulated bone formation later than rhBMP-2. This was observed from the quality of repaired bone tissue at four weeks following implantation. rhBMP-2 may directly stimulate the progenitor cells and osteoblasts; however, simvastatin needs to stimulate endogenous expression of BMP-2 first (13). Therefore, the simvastatin-loaded CS group showed delayed effects compared with the rhBMP-2-loaded CS group at four weeks following implantation, which was consistent with a previous study (29). However, no significant difference of new bone area between rhBMP-2-loaded CS group and simvastatin-loaded CS group was found at four and eight weeks following implantation. Furthermore, in accordance with a study by Mundy *et al* (13), no serious side effects were observed in the present study. Thus, the effect of simvastatin on bone repair was comparable with that of rhBMP-2, which may provide important information on its application. rhBMP-2 is an expensive substance, while simvastatin is inexpensive, approved worldwide, well tolerated and has been demonstrated to have a convenient side-effect profile (30). Thus, simvastatin may be used in the clinic to improve bone regeneration instead of, or in combination with rhBMP-2.

In conclusion, simvastatin may be efficiently released from simvastatin-loaded CS and induce osteogenic differentiation of MSCs. In addition, the advantages of simvastatin and desirable effects of rhBMP-2 on segmental bone repair were successfully combined with an efficient local application. Compared with rhBMP-2, simvastatin may be considered a cheap, well-tested drug with a beneficial side effect profile, and therefore, may be a promising substance in terms of bone regeneration. The simvastatin-loaded CS scaffold may have great potential in bone tissue engineering.

## Figures and Tables

**Table I:** Lane-Sandhu X-ray scores.

Indicator scores	X-ray scores
New bone formation	
None	0
<25%	1
25–50%	2
50–75%	3
>75%	4
Recreation of the marrow cavity	
No recreation	0
Partial recreation of marrow cavity	2
Cortical bone formation following recreation of marrow cavity	4
